# The Fourth Dimension of the Quadruple Aim: Empowering the Workforce to Become Partners in Health and Care

**DOI:** 10.5334/ijic.5985

**Published:** 2021-06-22

**Authors:** K. V. Stein, V. E. Amelung, R. Miller, N. Goodwin

**Affiliations:** 1 Department of Public Health and Primary Care, Leiden University Medical Centre, The Netherlands; 2 International Journal of Integrated Care, UK; 3 Institute for Social Medicine, Epidemiology and Health Systems Research, Medical University Hannover, Germany; 4 Global Engagement for College of Social Sciences, University of Birmingham, UK; 5 Central Coast Research Institute, Faculty of Health and Medicine, University of Newcastle and Central Coast Local Health District, AU

**Keywords:** workforce, education and training, people-centred systems, integrated care competencies

In 2014 Bodenheimer and Sinsky [1] proposed to expand the Triple Aim concept, and add a fourth dimension to it: improving the work life of professionals. They were not the first to draw attention to the sometimes precarious working conditions of health and care workers and the lack of strategic planning of decision makers to address an increasingly lopsided picture, where workforce migration, burn out and job dissatisfaction lead to an uneven availability in various professions (e.g. GPs, social workers), by country (migration from South to North and East to West) or region (rural-urban divide). The WHO has a long tradition of calling for action on the subject and, in the light of the Sustainable Development Goals (SDGs) and the COVID-19 pandemic, has declared 2021 the Year of the Health and Care Workers. They estimate that by 2030, there will be a global shortfall of 18 million health and care workers [2]. And while the reasons are complex, and the shortfall disproportionately hits low- and middle-income countries, the issues around it affect every health and care system in their ability to address population needs and build resilient communities.

As with so many other issues, long apparent and still overlooked by health and care systems, the pandemic has also put into high relief the plethora of challenges service providers are faced with every day: from the inability of sharing data adequately to the lack of communication across organisations and sectors, the power struggles between different professional groups to budget constraints and counterproductive incentives, to name but a few. And while the heroism and dedication of the health and care workforce has often been praised over the last months, few steps have yet been taken to ensure that we all come out stronger by addressing the challenges systemically and systematically.

“…health practitioners are people, and health care organizations and systems are made up of people. Their needs should also be considered, and they must be empowered to change the system for the better” [3].

When we talk about people-centred, integrated systems, creating the conditions to partner with people with lived experience, families and communities is only one side of the coin. We also need to involve service providers across all professions and sectors to co-design processes and organisations, which enable, motivate and inspire them to deliver people-centred care. Too often have we omitted to include the workforce’s perspective in the planning of integrated care initiatives, and ignored the fact that it’s people who need to work differently, act differently and use the new tools and technologies provided. As with everything else in integrated care design and implementation, none of the challenges are new or come as a surprise. They have been identified in the literature as key barriers to integration and universal health coverage for a long time, and over and over again. The need to change financing and incentives, education and training of the workforce, professional and organisational cultures, leadership and management, governance and accountability, etc. – all of these elements of an integrated, people-centred system also support and empower the workforce to become true partners in care with people, families and communities. What the pandemic offers us now is the opportunity to finally act on all levels and create the systemic framework and environment necessary for people to thrive on the ground.

## It all starts with trusted relationships

Learning by doing is certainly a crucial element in integrated care implementation, but there are lessons, which can be transferred from the vast experience gathered over the last decades. One of the most important ones being that usually the time necessary to involve, include and bring people on board takes a lot longer than anticipated – time that is often underestimated in the project plan, if it is calculated at all. But there are many examples from around the world, where projects and teams started off with a lot of steam and enthusiasm, before implementation stalled and after 6-12 months they had to return to the start, hit pause or abandon the efforts because they had overlooked that no one will want to share accountability and work across organisations and sectors, if they don’t trust their partners. Investing time and effort in building trusted relationships, providing formal and informal opportunities to meet, discuss differences, voice opposition, and thus build a common understanding und trust, will ultimately pay down the line when it comes to the nitty-gritty of implementation.

## Creating a supportive environment

Rebuilding trust is especially relevant now, as many frontline workers have lost trust in decisionmakers, managers and leaders considering the lack of leadership and mismanagement displayed in many countries. In a recent workshop, held during ICIC21, this frustration of the workforce was voiced, highlighting the need to actively invest in a new, shared and distributed approach to leadership and management. But creating a supportive environment for people and communities to thrive in necessitates more than a collaborative approach. The principles and values of a people-centred, integrated system need to be incorporated into accreditation mechanisms of professional bodies, the curricula of universities and the recruitment and continuous learning programmes of employers across sectors [4]. Governments and employers need to ensure that the new ways of working are supported by adequate incentives and regulatory frameworks, enough time, space and resources, as well as an outcomes-based monitoring system, which promotes and rewards team-based care giving and cross-sectoral collaboration, rather than limiting it to the absolute minimum of what is possible.

## Competencies for integrated care

The authors and this Journal have long called for a focus on the competencies of integrated care – and a better definition of what these competencies are (e.g. Stein 2016, Miller and Stein 2020 [5,6]). Asking people to work together in teams and collaboratively with people and communities requires a very different skill set to the specialised, disease and task-oriented delivery of services taught and practiced throughout the system. Integrated care does not come naturally to people, otherwise it would have developed long ago [7]. So, supporting the workforce also means changing the education and training systems to reflect this new way of working, and to emphasise the understanding that each and everyone has their role to play in a bigger system, and that no one “produces recovery and cure” singlehandedly. On the contrary, health and wellbeing are influenced by so many different factors that the more professions and sectors we include in the discussion, design, planning and delivery of health and care services, the better. Everyone needs to have a basic understanding of the social determinants of health and the roles and responsibilities of the people and professionals involved. But it also means that everyone needs to realise they are a peer learner, teacher and mentor as well. The way we treat families, other professionals and colleagues reflects the culture we practice in, and students, trainees and new colleagues will adopt that culture. There is no excuse for not at least reflecting on our own attitudes and values, even if we feel we cannot influence the system.

## The helping hand is female – and often has a migrant background

There are two interrelated aspects, which have so far been overlooked in integrated care literature, although it is well established in health services research and economics: 70% of the health and care workforce are women; and in high-income countries many of them have a migrant background [2,8]. And while, looking at individual professions, this percentage is even higher in social care, home care or nursing, it becomes increasingly true for medicine as well. This has profound effects on workforce planning, as women want more flexible working conditions, and the possibility of combining work life with family life. But it also reflects on the low value, bad image, and precarious work and pay conditions many of the health and care professions still face. It says a lot about society in high-income countries, when those who take care of the most vulnerable and whose daily work needs a lot of empathy, physical and mental strength, are among the least esteemed, worst paid and invisible. On the other hand, it leaves low- and middle-income countries with an even greater shortage of professionals on all levels of qualifications. If integrated, people-centred care subscribes to a more equitable health and care system, this must include a discussion around workforce planning, recruitment and distribution, not only within a country but across the North-South divide.

## Moving towards the Quadruple Aim – working as partners in care

The fatigue after 14 months of the pandemic is palpable, so it is of paramount importance to ensure that the workforce feel optimally supported in not only coping with the long-term effects but also with the move towards a more people-centred, integrated system of care. In our editorial from last summer [9], we already summarised some key learnings from the pandemic, one of them being that hierarchies and power dynamics have changed. So has the attitude towards working in teams and with a distributed leadership structure. In order to sustain these changes, a paradigm shift is necessary, across professions, systems and society to think and value health and wellbeing differently. This will be enabled by using the new power of networks, communities and value-driven people and harnessing their ideas for changing cultures, organisations and systems. The development of a workforce capable of delivering high-quality, people-centred and integrated care needs to be a priority on all levels, and competencies need to be the basis for planning, education and training, performance measurement and evaluation for integrated care. This is not only the right thing to do, it also makes economic sense. Personnel costs are the biggest cost unit in most organisations, and high fluctuation rates, a high number of sick days, or employees lacking the necessary competencies, all lead to even higher direct and indirect costs and suboptimal outcomes.

So, now is the time to reimagine the organisation of our systems, and building on the lessons learned so far: to use the immense potential of digital health and technologies strategically and make them the basis of service provision, from fostering health literacy to supporting long-term care; to build healthy communities, allow them to organise care close to home and redefine what primary care means; and to enable professionals to deliver the services necessary in partnership with their communities, and in care teams, which include volunteers and community members. Only then will we be able to realise our vision of a people-powered, integrated health and care system.
